# Yolk steroids in great tit *Parus major* eggs: variation and covariation between hormones and with environmental and parental factors

**DOI:** 10.1007/s00265-016-2107-1

**Published:** 2016-04-20

**Authors:** C. M. Lessells, S. Ruuskanen, H. Schwabl

**Affiliations:** Department of Animal Ecology, Netherlands Institute of Ecology (NIOO-KNAW), Droevendaalsesteeg 10, PO Box 50, 6700 AB Wageningen, The Netherlands; Section of Ecology, Department of Biology, University of Turku, Turku, Finland; Center for Reproductive Biology, School of Biological Sciences, Washington State University, Pullman, WA 99164 USA

**Keywords:** Yolk steroids, Avian, Birds, Ambient temperature, Incubation, Hatching asynchrony

## Abstract

**Abstract:**

Avian mothers can potentially alter the phenotypes of their offspring by varying the concentration of steroid hormones in their eggs. We explored variation in androstenedione (A4), testosterone (T), 5α-dihydrotestosterone (DHT), 17β-estradiol (E2), and corticosterone (CORT) in the yolks of 12 free-living great tit *Parus major* clutches. We analyzed variation and covariation in greater detail than previous studies, using models for variation with laying sequence that take into account variable clutch size and comparing correlations between pairs of hormones at the within- and between-clutch levels. We also investigated relationships between hormone levels and various environmental, life history, and parental traits. For three of the five steroids, we found no significant correlates, but based on individual statistical tests (a) DHT varied between clutches with male age (1 year old vs older); (b) DHT and CORT were negatively correlated within clutches with the average temperature on the day (DHT and CORT) or 3 days (DHT only) preceding laying; and (c) DHT in the last egg of the clutch relative to the clutch mean was positively correlated with the interval between clutch completion and the onset of incubation (*incubation delay*). Relationships with ambient temperature and incubation delay have not previously been reported for any yolk hormone in birds. Intriguingly, the three relationships for DHT are consistent with more DHT being transferred to eggs in situations that could be more energetically challenging for the female. More research is needed to determine the generality of the patterns we found and to understand their functional significance.

**Significance statement:**

The yolks of birds’ eggs contain steroid hormones produced by the mother which can affect the development and behavior of the resultant chicks. We analyzed five steroid hormones in the yolks of wild great tits and show for the first time that yolk hormone levels are related to ambient temperature in the day(s) just before laying and, in the last-laid egg, with the day it is laid relative to the onset of incubation, and that the concentrations of pairs of yolk hormones can vary with each other in a different way between and within clutches. These results contribute insights into the ways in which yolk hormones may adaptively modify the chicks or may reflect physiological processes occurring in the mother.

**Electronic supplementary material:**

The online version of this article (doi:10.1007/s00265-016-2107-1) contains supplementary material, which is available to authorized users.

## Introduction

The discovery that avian egg yolks contain maternally derived steroid hormones (Schwabl 1993) has ignited interest in the significance and function of variation in the amount of maternal hormones transferred to eggs (Groothuis et al. 2005; Gil 2008). Yolk hormones interest evolutionary and behavioral ecologists and physiologists for several reasons: first, hormone-mediated maternal effects provide a means for the female to modify offspring phenotype (from growth up to adult phenotype and behavior) in anticipation of environmental conditions (Schwabl et al. 1997; Groothuis et al. 2005), thus making them a good model to study adaptive trans-generational phenotypic plasticity. Conceptualized in this way, variation in yolk hormone levels impacts offspring fitness and is in the interests of both mother and offspring. Second, if females are unable to adjust yolk hormones independent of the levels of circulating hormones, the amount of hormones transferred to eggs should reflect the balance between the effects of yolk hormones on offspring fitness, and circulating hormones on maternal fitness (e.g., Müller et al. 2007; Tobler and Smith 2010). When the former predominates, adaptive trans-generational phenotypic plasticity is indeed expected, but if the latter predominates, variation in yolk hormones will tend instead to reflect the functional significance of hormones in the mother (e.g., regulating the onset or termination of laying or incubation) rather than adaptive effects on the offspring. One of the key questions is therefore whether females are able to adjust yolk steroids independent of the levels in their own circulation (Groothuis and Schwabl 2008; see also Müller et al. 2011; Egbert et al. 2013). Lastly, relatedness asymmetries within the family may result in evolutionary conflicts in which the genetic interests of family members do not entirely coincide. By offering a means of manipulating sibling competition and begging behavior in chicks, maternally derived yolk hormones might enable the mother to impose her individual optimum in terms of the chicks’ behavior and development or to induce higher levels of investment by the male parent and hence win out in parent-offspring and sexual conflict, respectively (Lessells 2006; Müller et al. 2007; Horváthová et al. 2012).

Attempts to understand the relative importance of the above processes have focused on both within- and between-clutch variation. Within clutches, increasing or decreasing concentrations of yolk hormones through the laying sequence have often been interpreted in relation to hatching asynchrony, which produces a size hierarchy among nestlings resulting in later-hatching chicks being at a competitive disadvantage. The “hormonal parental favoritism” hypothesis suggests that variation in yolk androgens within the laying sequence mitigates or accentuates the effects of hatching asynchrony (e.g., Schwabl 1993; Schwabl et al. 1997; Groothuis et al. 2005; Müller and Groothuis 2013). Alternatively, within-clutch variation might reflect the involvement of hormones in maternal physiology and “leakage” into developing yolks (Groothuis and Schwabl 2008). The match between patterns of variation in hormone concentrations in the clutch and patterns of hatching asynchrony on the one hand, and maternal physiological processes on the other, may help to distinguish the above possibilities.

Between-clutch variation substantially exceeds within-clutch variation in most species (Groothuis et al. 2005) and could represent facultative adjustment of offspring phenotype to anticipated conditions or reflect the female’s hormonal state. Variation among clutches in yolk androgens has been found to relate to various environmental and social conditions such as food supply (Verboven et al. 2003; Sandell et al. 2007), breeding density (Pilz and Smith 2004; Remeš 2011), female social interactions (Whittingham and Schwabl 2002), condition (Pilz et al. 2003; Tobler et al. 2007), social status (Müller et al. 2002; Tanvez et al. 2008), and exposure to parasites (Tschirren et al. 2004). Variation in androgen concentrations in eggs in relation to male attractiveness (e.g., plumage coloration, song) or age might reflect differential allocation on the part of females or an attempt to manipulate their mate’s parental investment (Groothuis et al. 2005; but see Horváthová et al. 2012).

The majority of research so far has involved androgens, especially androstenedione and testosterone, but recently, corticosterone, estradiol, and thyroid hormones have been investigated (McNabb 2007; Groothuis and Schwabl 2008; Love et al. 2009). We would like to emphasize that there is no a priori justification for the intense focus on testosterone in this research: neglecting yolk 5α-dihydrotestosterone, a biologically potent androgen with very high affinity for the androgen receptor (Groothuis and Schwabl 2008) or 17β-estradiol (important in orchestrating female reproductive physiology and offspring sexual differentiation, e.g., Williams 1999; Williams et al. 2005) may give a misleadingly incomplete picture. Moreover, these steroids constitute a synthesis pathway from androstenedione to testosterone to 5α-dihydrotestosterone or 17β-estradiol. Corticosterone (the major avian glucocorticoid and stress hormone) in eggs varies in relation to the mother’s plasma levels and stress and is known to affect offspring phenotype, including growth, begging, and immunity (Hayward and Wingfield 2004; Love et al. 2005; Saino et al. 2005; Okuliarova et al. 2010; Schoech et al. 2011; Almasi et al. 2012). As for androgens, estradiol and corticosterone may vary within clutches (e.g., Williams et al. 2005; Love et al. 2009; Rubolini et al. 2011), but further studies are needed to reveal general patterns.

In this study, we explore within- and between-clutch variation in the concentration of five steroids-androstenedione (A4), testosterone (T), 5α-dihydrotestosterone (DHT), 17β-estradiol (E2), and corticosterone (CORT)-in the egg yolks of free-living great tits *Parus major*. In particular, we examine (1) hormone concentrations, their repeatability, and how they vary through the laying sequence. We predict that, if variation in yolk steroids with laying sequence is mainly related to exaggerating or mitigating the effects of hatching asynchrony, the hormone levels of the last egg should be most different from the rest of the clutch, with little systematic variation across earlier laid eggs. This is because diurnal incubation of first clutches in the study population rarely begins more than 1 day before clutch completion (3/38 first clutches in 1999, with no full incubation initiated more than 2 days before clutch completion), resulting in hatching asynchrony being low and concentrated on the last laid egg. We further examine (2) between- and within-clutch variation in relation to environmental, life history, and parental characteristics and (3) the relationship between concentrations of steroids in the last egg of the clutch relative to the other eggs and the interval between clutch completion and the onset of incubation. We predict that such a correlation will exist if steroids are involved in the regulation of the onset of incubation and incidentally diffuse into the developing yolks (reviewed, e.g., in Williams et al. 2005; Sockman et al. 2006). In all analyses, except those for correlations between the concentrations of different hormones (which we analyze in a pair-wise fashion), we analyze data separately for each hormone and provide estimates from models with single explanatory variables, in order to allow comparisons with current and future studies, including meta-analyses. We are aware of the risks related to multiple testing and have controlled for inflated *P* values using a false discovery rate (FDR) approach (Benjamini and Hochberg 1995; Benjamini 2010). The analyses that we report here are novel in a number of respects: first, we consider a greater range of steroids than previous studies. This allows us to investigate the correlations between a larger number of pairs of steroids and to demonstrate for the first time that correlations between steroids may differ in strength at the within- and between-clutch levels. Second, we investigate previously neglected correlates and reveal for the first time within-clutch relationships between steroids and the temperature preceding laying, and the levels of a steroid in the last egg of the clutch in relation to the delay between clutch completion and the onset of incubation. These are important contributions in a field where, due to the predominance of experiments, the patterns and correlates of natural yolk hormones are poorly known.

## Methods

### Collection of yolks and parental data

Eggs were collected in April 1999 from a nest box population of great tits at Westerheide, a mixed woodland in the Veluwe, The Netherlands. Eggs were generally collected on the day of laying, and each egg removed was replaced with a dummy egg. Eggs were collected from 38 clutches that were inspected daily until 2 days after clutch completion or until incubation began (whichever was later). Mean daily temperature (mean of 24 hourly measurements) was obtained from the Royal Dutch Meteorological Institute (KNMI) (see Lessells et al. 2002 for further details of field data collection and temperature data). By chance, temperature dropped markedly during the middle of the period when great tits were laying in 1999 (see Lessells et al. 2002, Fig. 1c), so that temperature and laying date were correlated between (hierarchical mixed model (see “Methods”), *b* = 0.353 °C/day, *F*_1,9.92_ = 17.39, *P* = 0.002), but not within (*b* = −0.011 °C/day, *F*_1,80_ = 0.02, *P* = 0.875), clutches, allowing the effects of temperature to be statistically separated from those of laying date (and sequence) within, but not between, clutches. Yolk hormone levels were analyzed from 12 clutches where incubation was begun, and for which the most complete data on the parents were available. These clutches did not differ in laying date from the collected clutches that were not analyzed [yolk hormone levels analyzed, laying date = 17.92 (April) ± 1.86 (SE) days, range = 7–25 (April); not analyzed, 18.34 ± 0.99, range = 8–24; *F*_1,36_ = 0.05, *P* = 0.824]. Eggs were opened in the laboratory on the day of collection and the yolks separated from the remainder of the egg. Excess albumen was removed from the yolk by rolling it on a piece of paper, and the yolk frozen at −20 °C until hormone analysis. Mass (to the nearest mg) was obtained for the whole egg, wet yolk, wet albumen, and dry shell (see Lessells et al. 2002 for further details). Breeding adults were identified from color bands or the females caught on the nest during incubation (before their dummy clutch was removed). Parental age was determined from their banding history or from plumage characters, and parent birds were classified into 1-year-old and older categories for analysis. The 12 pairs included four 1-year-old males and six 1-year-old females, and there were two nests where both parents were 1 year old. Tarsus length of both parents was measured to the nearest 0.1 mm using vernier callipers, either when they were originally caught for banding or when caught during the current study. It was not possible to record data blind because our study involved focal nests and individuals in the wild.

**Fig. 1:**
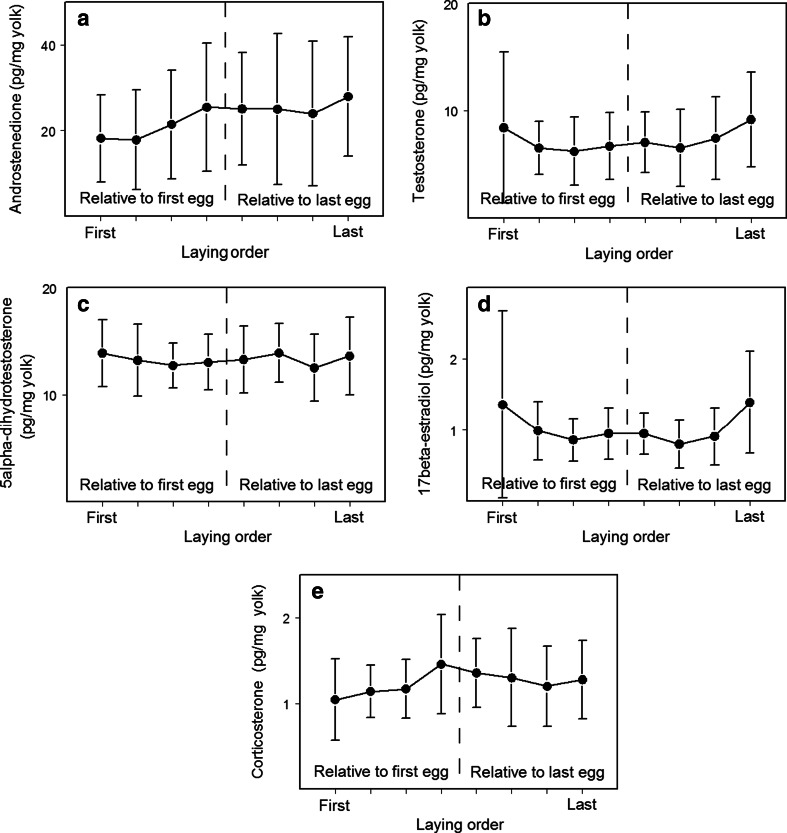
Mean concentrations (±SE) of **a** A4, **b** T, **c** DHT, **d** E2, and **e** CORT in the yolks of great tit eggs in relation to laying sequence (*n* = 12 clutches). Means and SEs are based on untransformed values. Laying order is expressed relative to the first egg in the clutch for the first four eggs and the last egg in the clutch for the last four eggs. Because clutch size was not always exactly eight, seven eggs (in clutches of 6 or 7 eggs) contribute to more than one mean, while four eggs (in clutches of 9 and 11 eggs) do not contribute to any mean. This was done to give a visual representation of variation in hormone levels in relation to the onset and termination of laying. However, because few eggs were omitted or represented twice, the figures are a good approximation to the relationship with the egg number. We emphasize that all statistical analyses were carried out in a conventional manner with individual eggs included once (and only once) in each analysis

### Hormone analysis

We measured A4, T, DHT, E2, and CORT in the yolks of 93 eggs (91 for CORT) from 12 great tit clutches. Whole yolks were homogenized in distilled water (1 μl/mg) with the aid of the addition of glass beads. On average, 250 mg of this yolk/water emulsion was transferred to glass test tubes and extracted twice with 4 ml of petroleum ether/diethyl ether (30:70 *v*:*v*). Combined extracts were dried under nitrogen and re-suspended in 1 ml of 90 % ethanol. After overnight precipitation of lipids and proteins at −20 °C, extracts were centrifuged and the supernatant was collected and dried. Steroids were separated and further purified using diatomaceous earth column chromatography. Hormone fractions were dried and then dissolved in 560 μl of phosphate-buffered saline (with gelatine). Duplicates of 200 μl were used in standard radio-immunoassays, and 100 μl was used to estimate recovery. Mean recoveries were as follows: A4 = 66 %, DHT = 42 %, T = 55 %, E2 = 57 %, and CORT = 61 %; the hormone concentrations of each sample were corrected by individual recoveries. The following tritiated steroids from New England Nuclear were used: A4 (NET-469), DHT (NET-463), T (NET-553), E2 (NET-517), and CORT (NET-399). Polyclonal antibodies used were the following: A 1707 for A4 and T 3003 for T and DHT (Wien Laboratories, Inc., Succasunna, NJ), E 1702 (*Arnel*, New York, NY) for E2, and B3-163 (Esoterix Endocrinology, Inc.) for CORT. The CORT antibody cross-reacts with progesterone which was, however, separated from the CORT fraction by chromatography. We performed a single assay for each hormone with clutches randomized, but the eggs of clutches kept together. The assays had the following intra-assay variation: A4 = 5.5 %, DHT = 7.2 %, T = 6.1 %, E2 = 5.1 %, and CORT = 4.8 %.

### Statistical analysis

The distribution of all hormone concentrations except DHT (*P* = 0.54, skewness = −0.240) departed significantly from normality (all *P* < 0.0001) and were right-skewed (skewness values in the range 1.063–2.733). For the four hormones that were not normally distributed, log transformation removed (A4, E2) or reduced (T, CORT) the departure from normality and also reduced skewness. Moreover, except for DHT (*r* = −0.29, *N* = 12, *P* = 0.37), the standard deviation of hormone concentration was significantly positively correlated across clutches to the mean of the clutch (*r* = 0.62 to 0.94, *N* = 12, *P* = 0.034 to <0.001). For the four hormones that were not normally distributed, log transformation removed (A4, T, E2) or reduced (CORT) this correlation. Unless otherwise stated, the analyses below were carried out on untransformed values of DHT, but log-transformed values of A4, T, E2, and CORT. Repeatability values and their standard errors were calculated from linear mixed-effects models fitted using REML with the package rptR (R Core Team 2013) in R version 3.0.2.

Analyses of hormone concentrations in relation to within- and between-clutch variation in a range of explanatory variables were carried out using hierarchical mixed models in PROC MIXED of SAS version 8.2 (Singer 1998; SAS Institute Inc. 2009). The clutch to which eggs belonged was treated as a random effect in all models, and the variance due to clutch was significantly greater than zero in all models reported below. *P* values reported for fixed effects are type III (*unique*); that is, they are the *P* values for inclusion (non-significant effects) or exclusion (significant effects) of the variable from the final model. Fixed effects can potentially act at the level of either the clutch (variables such as clutch size or parental characteristics, which are the same for all eggs in a clutch) or at both the egg and clutch level. Yolk mass is an example of an effect that can act at both levels: the concentration of a hormone in an individual egg may be related to the yolk mass of that egg, and the mean concentration of a hormone in a clutch may be related to the mean yolk mass of the clutch. Such an effect can work independently at the two levels (i.e., exist at one level and not the other). This sort of effect was tested in the model by including both the clutch mean value of the predictor variable and the individual values of the same variable expressed as deviations from the clutch mean (*centered* values) in the model (Singer 1998; van de Pol and Wright 2007) and using Satterthwaite’s method for estimating the denominator degrees of freedom. As always in interpreting the results of regression (and correlation) analyses, it should be remembered that a significant relationship does not imply causation (or the causal direction) between the response and explanatory variables. The analysis is descriptive, and the terms “response” and “explanatory” refer to statistical, and not causal, explanation.

We analyzed correlations between the concentrations of pairs of hormones using hierarchical mixed models in PROC MIXED of SAS version 8.2. As for the above analysis that analyzes the concentrations of single hormones in relation to within- and between-clutch variation in a range of explanatory variables, correlations between pairs of hormones can differ at the between- and within-clutch levels. We estimated the correlation coefficients at these two levels and attached *P* values using likelihood ratio tests following Dingemanse and Dochtermann (2013, Supplementary Material, pages 29 and 46). Differences in correlation coefficients at the two levels are of interest because they would indicate that different extrinsic or intrinsic factors are at play at the two levels. The number of clutches we sampled was too small to test this difference meaningfully using mixed models (Yimen Araya-Ajoy and Niels Dingemanse, personal communication). We therefore tested whether the Pearson correlation coefficients between the clutch means and between the centered values of pairs of hormones differed using a *t* test for unequal variances (Welch’s *t* test) with the degrees of freedom estimated using the Welch-Satterthwaite method (Wikipedia 2014). The correlation between clutch means is not an unbiased estimate of the between-clutch correlation, but its use in this context is conservative as it is a weighted average of the between- and within-clutch correlations (see Online Resource 1).

Modeling laying order effects in a species like the great tit is complicated by the variation in clutch size. Biological hypotheses (both in terms of the fitness consequences for offspring and hormonal control in the mother) imply that the first or last eggs in a clutch may differ from the others in addition to any trend through the clutch. In this circumstance, analyzing hormone levels in terms of only egg number (e.g., Reed and Vleck 2001) is inadequate, because with variable clutch size, the last egg will fall into a range of different egg number categories. We therefore modeled hormone concentrations by fitting three fixed effects: the centered egg number (individual egg number − average egg number in the clutch), which models a linear trend (positive or negative) through the laying order, and variables for the first and last eggs in the clutch, modeling positive or negative departures from the overall linear relationship for each of these two eggs. Initially, all three fixed effects were included in the model, and non-significant effects dropped sequentially. For comparison with other studies, we also report the results of analyses using “proportional laying order” (which scales position in the laying order in each clutch linearly from 0 for the first egg to 1 for the last egg) and its square. In three clutches, the laying order within two or three eggs in the clutch was unknown, and these eggs were excluded from the analyses involving laying order.

Lastly, because we have carried out multiple statistical tests for similar hypotheses, we have controlled for inflated *P* values using a FDR approach (Benjamini and Hochberg 1995; Benjamini 2010). The FDR is the expected proportion of type I errors among all significant results. We controlled the FDR to a family-wise probability of less than 0.05. We report the *P* values for individual tests and indicate values that become *non-significant* when controlling FDR (i.e., for which FDR > 0.05).

## Results

Hormone concentrations were measured in the yolks of 93 eggs (91 for CORT) from 12 complete clutches (see Online Resource 2 for data). Clutch size ranged from 6 to 11 eggs (mean = 7.75 ± 0.94 (SE)). Mean hormone concentrations are given in Table 1. Average concentrations of A4, T, and DHT were close to those reported in other studies of great tits (e.g., Tschirren et al. 2004; Groothuis et al. 2008; Remeš 2011). For E2 and CORT, other studies on great tits (Tschirren et al. 2004; Groothuis et al. 2008) and other species (e.g., Love and Williams 2008) report similarly low (or undetectable) levels.

**Table 1:** Concentrations and repeatabilities of A4, T, DHT, E2, and CORT in the yolks of great tit eggs

	Hormone concentration (pg/mg yolk)		*F* (*df*)^a, b^	Repeatability ± SE^b^
	Mean ± SE (*N*)^c^	Range in clutch means		
A4	23.35 ± 1.423 (93)	7.89–44.85	22.5 (11,81)	0.727 ± 0.105
T	7.22 ± 0.411 (93)	1.92–11.93	10.9 (11,81)	0.560 ± 0.129
DHT	13.07 ± 0.317 (93)	9.83–16.09	4.15 (11,81)	0.291 ± 0.117
E2	1.063 ± 0.070 (93)	0.345–1.633	6.15 (11,81)	0.391 ± 0.128
CORT	1.258 ± 0.045 (91)	0.858–1.756	6.28 (11,79)	0.425 ± 0.128

^a^ *F* ratios are for one-way ANOVAs between clutches; all *P* < 0.0001. Controlling the FDR at *P* < 0.05, all repeatability values remain significant

^b^ *F* ratios and repeatabilities are based on log-transformed values for all hormones except DHT

^c^Means and SEs are based on untransformed values

All five hormones were significantly repeatable within clutches (Table 1). Repeatability (across the eggs within a clutch) ranged from 0.291 for DHT to 0.736 for A4. Low repeatability, such as that of DHT, in general implies that more eggs must be sampled per clutch to obtain an adequate estimate of the mean level of that hormone in the clutch. Mean yolk mass was 315.7 ± 2.68 (SE) mg and also varied significantly among clutches (repeatability = 0.491 ± 0.127, *F*_11,81_ = 8.61, *P* < 0.001; see also Lessells et al. 2002).

### Correlations among yolk hormones between and within clutches

Relationships between hormones at either the level of the clutch or individual egg were investigated in pair-wise analyses of hormones (Table 2; Online Resource 1). All of the significant correlations at either the between- or within-clutch level between different hormones were positive. In general, between-clutch correlation coefficients tended to be higher than within-clutch coefficients (although the pattern of significance is sometimes reversed because of the differences in sample size at the two levels). T and E2 were highly correlated (*r* > 0.9) both within and between clutches. T and E2 were each also highly correlated (*r* > 0.8) between clutches with A4, but the within-clutch correlation of each of these hormones with A4 was significantly lower (T: *P* = 0.002, E2: *P* = 0.004; see Online Resource 1, Table OR1.3) and was only significantly greater than zero for the relationship between A4 and T. The estimated between-clutch correlation coefficients are higher than the within-clutch coefficients for DHT with A4 or T, but these differences are not significant (A4: *P* = 0.658, T: *P* = 0.161; see Online Resource 1, Table OR1.3) and only the within-clutch correlation coefficient between A4 and DHT differs significantly from zero. For correlations involving CORT, the only significant correlations were the between-clutch correlations with T and E2, and the latter becomes non-significant after controlling FDR. These two correlations should be treated with caution, because visual inspection of the relationships between the clutch means suggests that the correlations are largely due to a single clutch with low mean concentrations of T, E2, and CORT (see Online Resource 1, Fig. OR1.1m and s).

**Table 2:** Between- and within-clutch correlations between A4, T, DHT, E2, and CORT in the yolk of great tit eggs

	*r*(*P* ^a^) [upper value is between-clutch *r* ^b^ (*n* = 12); lower value is within-clutch *r* ^a^ (*n* = 93, except for correlations involving CORT, where *n* = 91)]			
	Log(A4)	Log(T)	DHT	Log(E2)
Log(T)	*0.903* (*<0.001*)			
	*0.274* (*0.011*)			
DHT	0.422 (0.273)	0.669 (0.065)		
	*0.279* (*0.010*)	0.153 (0.168)		
Log(E2)	−(^c^)	0.925 (0.025)	0.600 (0.061)	
	0.153 (0.168)	*0.906* (*<0.001*)	−0.019 (1.000)	
Log(CORT)	0.315 (0.061)	*0.502* (*0.009* ^d^)	0.025 (0.752)	0.531 (0.043^e^)
	0.210 (0.058)	−0.043 (0.655)	0.179 (0.107)	−0.118 (0.294)

^a^ *P* values are from individual statistical tests. FDR was controlled in separate families consisting of the ten *P* values for between-clutch correlations and the ten *P* values for within-clutch correlations. Italicized values indicate FDR < 0.05

^b^Between- and within-clutch correlation coefficients (*r*) are estimated from mixed models (see “Methods”). *P* values are from likelihood ratio tests based on *χ* ^2^ with 1 *df*

^c^ *r* and *P* value for the likelihood ratio test are missing because estimation stopped after too many likelihood estimations. The *P* value for the Pearson correlation coefficient for the clutch mean hormone concentrations is <0.001 (see Online Resource 2, Table OR2.1)

^d^The *P* value for the Pearson correlation coefficient for the clutch mean hormone concentrations is 0.129 (see Online Resource 2, Table OR2.1)

^e^The *P* value for the Pearson correlation coefficient for the clutch mean hormone concentrations is 0.132 (see Online Resource 2, Table OR2.1)

### Yolk hormones in relation to the mass of the egg or egg components

Hormone concentrations did not generally vary significantly with the mass of the whole egg, wet yolk, wet albumen, or dry shell mass, at either the clutch or individual egg level (Table OR3.1). The only exception was CORT which increased significantly with shell mass within clutches (Table OR3.1), and this relationship was not significant after controlling FDR. The mass of the whole egg or that of egg components has therefore not been included as a covariate in the analyses of hormone concentrations.

### Yolk hormones in relation to the laying sequence

The concentrations of all hormones except DHT varied through the laying sequence (Fig. 1, Table 3). A4 increased linearly through the laying order. T was higher in the last egg of the clutch but otherwise did not vary with position in the laying sequence. CORT was lower in the first egg of the clutch but otherwise did not vary through the laying sequence. E2 decreased through the laying order, except for the last egg, which contained significantly more E2 than expected on the basis of the linear decrease. The patterns of variation in CORT and E2 can also be alternatively described by a quadratic relationship with the proportional laying order (Table 3).

**Table 3:** Concentrations (pg/mg yolk) of A4, T, DHT, E2, and CORT in the yolks of great tit eggs in relation to laying sequence: estimates are for models obtained by removal of non-significant terms from initial models containing (i) the centered egg number and terms for the first and last eggs in the clutch and (ii) the proportional laying order and its square

			Estimate ± SE	*F* (*ddf* ^a^)	*P* ^b^
Log(A4)	(i)	Intercept	*3.001*		
		Centered egg no.	*0.0343 ± 0.0132*	*6.79* (*73.3*)	*0.011*
		First egg^b^	[0.0084 ± 0.1150]	0.01 (72.1)	0.942
		Last egg^b^	[0.1471 ± 0.1051]	1.96 (72.1)	0.166
	(ii)	Intercept	*2.860*		
		Prop. laying order	*0.2761 ± 0.0928*	*8.85* (*73.4*)	*0.004*
		Prop. laying order^2^	[−0.0773 ± 0.3235]	0.06 (72.2)	0.812
Log(T)	(i)	Intercept	*1.804*		
		Centered egg no.	[−0.0153 ± 0.0211]	0.53 (72.6)	0.469
		First egg	[0.1424 ± 0.1315]	1.17 (74.5)	0.282
		Last egg	*0.2728 ± 0.1212*	*5.07* (*74.3*)	*0.027*
	(ii)	Intercept	*1.838*		
		Prop. laying order	[0.1141 ± 0.1274]	0.80 (73.9)	0.373
		Prop. laying order^2^	[0.1622 ± 0.1196]	1.84 (73.8)	0.179
DHT	(i)	Intercept	*13.155*		
		Centered egg no.	[−0.0650 ± 0.1171]	0.31 (74.5)	0.580
		First egg	[1.0840 ± 0.8860]	1.50 (76.1)	0.225
		Last egg	[0.5624 ± 0.8088]	0.48 (80.2)	0.489
	(ii)	Intercept	*13.155*		
		Prop. laying order	[−0.2076 ± 0.8358]	0.06 (75)	0.804
		Prop. laying order^2^	[0.1086 ± 0.7915]	0.02 (74.6)	0.891
Log(E2)	(i)	Intercept	*−0.1487*		
		Centered egg no.	*−0.0495 ± 0.0228*	*4.72* (*73*)	*0.033*
		First egg	[0.0338 ± 0.1703]	0.04 (71.6)	0.843
		Last egg	*0.5060 ± 0.1523*	*11.04* (*72.5*)	*0.001*
	(ii)	Intercept	*−0.0883*		
		Prop. laying order	[−0.0845 ± 0.1332]	0.00 (74.7)	0.997
		Prop. laying order^2^	[0.0746 ± 0.1358]	0.30 (74.4)	0.584
Log(CORT)	(i)	Intercept	*0.185*		
		Centered egg no.	[0.0089 ± 0.0137]	0.42 (70.3)	0.519
		First egg	*−0.2205*	*6.26* (*73.8*)	*0.015*
		Last egg	[−0.0073 ± 0.0785]	0.01 (72.2)	0.926
	(ii)	Intercept	*−0.0344*		
		Prop. laying order	*0.9253*	*9.70* (*70.8*)	*0.003*
		Prop. laying order^2^	*−0.7538*	*7.38* (*70.4*)	*0.008*

Reported values are from hierarchical mixed models with clutch as a random effect. The final models (estimates in italic) were obtained by successive removal of non-significant terms. Estimates and (type III) *P* values were obtained from the final model for significant terms and by addition of the variable into the final model for non-significant terms (for which the estimates are given in square brackets)

*ddf* denominator *df*

^a^All numerator *df* = 1

^b^First (last) egg has the value 1 for eggs that are first (last) laid in a clutch and 0 for other eggs

### Correlates of yolk hormones between and within clutches

**Fig. 2:**
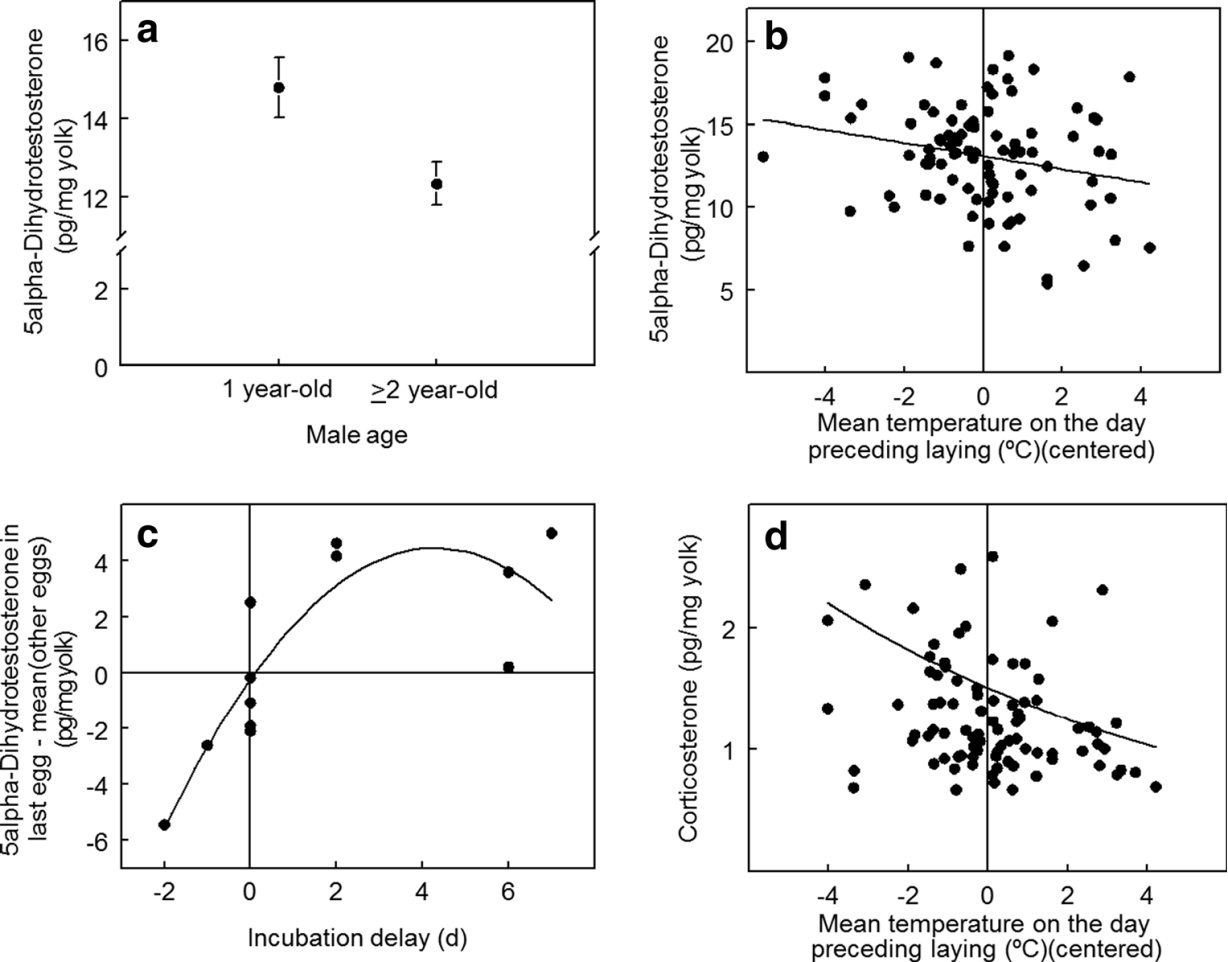
Between- and within-clutch correlates of hormones in the yolks of great tit eggs: **a** DHT (clutch mean ± SE) in relation to male age (between-clutch), **b** DHT in relation to the mean temperature on the day preceding laying (relationship with the mean temperature on the 3 days preceding laying also significant) (within-clutch), **c** DHT in the last egg of the clutch minus the mean of other eggs in the same clutch in relation to the incubation delay (= date of the onset of incubation − date of laying of last egg; negative vales of incubation delay indicate that incubation began before clutch completion) (within-clutch), and **d** CORT in relation to the mean temperature on the day preceding laying (within-clutch). Fitted curves in **b**, **d** are from hierarchical mixed models with clutch as a random effect, and only the *x*-axis variable fitted as an explanatory variable. The fitted curve in **c** is calculated as the equation given in Table 6 with last egg set to 1, minus the equation given in Table 6 with last egg set to 0. The resultant function is −0.226 + 2.186(incubation delay) − 0.255(incubation delay)^2^. The analysis of CORT in **d** was based on log-transformed values

Between clutches, hormone concentrations did not vary with clutch size, laying date of the first egg in a clutch, tarsus length of either parent, female age (Table 4), or temperature preceding laying (Table 5). However, DHT was significantly lower in the clutches of older males (Table 5, Fig. 2a; 1-year-old males = 14.79 ± 0.78 pg/mg; ≥2-year-old males = 12.33 ± 0.55 pg/mg), although this result became non-significant when controlling the FDR.

**Table 4:** Concentrations (pg/mg yolk) of A4, T, DHT, E2, and CORT in the yolks of great tit eggs in relation to life history and parental traits

	Between clutches			
	*b* ± SE	*F*	*ddf* ^a^	*P* ^b^
Explanatory variable: clutch size				
Log(A4)	0.0303 ± 0.1206	0.06	9.9	0.806
Log(T)	−0.0519 ± 0.1116	0.22	9.8	0.652
DHT	−0.5450 ± 0.4296	1.61	9.3	0.213
Log(E2)	−0.0111 ± 0.0927	0.01	9.7	0.442
Log(CORT)	−0.0191 ± 0.0570	0.11	9.4	0.744
Explanatory variable: laying date of the first egg in clutch				
Log(A4)	0.0139 ± 0.0239	0.34	10.0	0.572
Log(T)	0.0042 ± 0.0227	0.03	10.0	0.856
DHT	0.0305 ± 0.09376	0.11	10.0	0.751
Log(E2)	0.0037 ± 0.0187	0.04	10.1	0.845
Log(CORT)	−0.0137 ± 0.0107	1.66	9.6	0.228
Explanatory variable: male age^c^				
Log(A4)	0.098 ± 0.316	0.10	10.0	0.762
Log(T)	−0.074 ± 0.296	0.06	10.0	0.807
DHT	−2.461 ± 0.952	6.68	9.9	0.027
Log(E2)	0.123 ± 0.245	0.00	10.2	0.961
Log(CORT)	−0.047 ± 0.151	3.03	9.8	0.763
Explanatory variable: female age^c^				
Log(A4)	−0.482 ± 0.258	3.50	10.0	0.091
Log(T)	−0.325 ± 0.260	1.57	10.0	0.238
DHT	−1.441 ± 1.070	1.82	10.1	0.207
Log(E2)	−0.230 ± 0.219	1.10	10.1	0.318
Log(CORT)	0.119 ± 0.137	0.75	9.7	0.408
Explanatory variable: male tarsus length (mm)				
Log(A4)	0.0348 ± 0.3866	0.01	7.0	0.931
Log(T)	0.1979 ± 0.3563	0.31	7.0	0.596
DHT	−1.668 ± 1.482	1.27	6.8	0.298
Log(E2)	0.2690 ± 0.3130	0.74	7.0	0.419
Log(CORT)	0.1170 ± 0.1830	0.41	6.9	0.541
Explanatory variable: female tarsus length (mm)				
Log(A4)	−0.0484 ± 0.3822	0.02	10.0	0.902
Log(T)	−0.1495 ± 0.5360	0.18	9.9	0.681
DHT	−1.138 ± 1.432	0.63	9.8	0.445
Log(E2)	−0.0513 ± 0.2943	0.03	10.0	0.865
Log(CORT)	−0.0254 ± 0.1818	0.02	9.7	0.892

Reported values are from hierarchical mixed models with clutch as a random effect. Explanatory variables have the same value for all eggs within a clutch

*ddf* denominator *df*

^a^All numerator *df* = 1

^b^ *P* values are from individual statistical tests. FDR was controlled in a family consisting of the tests of between-clutch relationships for each steroid (all relationships in this table plus between-clutch relationships in Table 5)

^c^One-year-old (0) vs older (1)

^d^FDR > 0.05 for *P* values ≤ 0.05

**Table 5:** Concentrations (pg/mg yolk) of A4, T, DHT, E2, and CORT in the yolks of great tit eggs in relation to the mean temperature on the day or 3 days preceding laying of each egg

	Between clutches				Within clutches			
	*b* ± SE	*F*	*ddf* ^a^	*P* ^b^	*b* ± SE	*F*	*ddf* ^a^	*P* ^c^
Explanatory variable: mean temperature on the day preceding laying								
Log(A4)	0.0338 ± 0.0577	0.34	10.0	0.571	−0.0056 ± 0.0178	0.10	77.1	0.756
Log(T)	0.0121 ± 0.0549	0.05	10.1	0.829	−0.0142 ± 0.0234	0.37	77.1	0.547
DHT	−0.0009 ± 0.2284	0.00	10.1	0.997	*−0.3954 ± 0.1483*	*7.10*	*77.3*	*0.009*
Log(E2)	−0.0022 ± 0.0457	0.00	10.1	0.963	−0.0134 ± 0.0262	0.26	77.3	0.612
Log(CORT)	−0.0170 ± 0.0263	0.42	9.7	0.534	*−0.0413 ± 0.0159*	*6.73*	*76.1*	*0.011*
Explanatory variable: mean temperature on the 3 days preceding laying								
Log(A4)	0.0479 ± 0.0678	0.50	10.0	0.496	0.0270 ± 0.0171	2.50	77.1	0.118
Log(T)	0.0297 ± 0.0643	0.21	10.0	0.654	0.0074 ± 0.0228	0.09	77.2	0.747
DHT	0.0007 ± 0.2698	0.00	10.1	0.998	*−0.3400 ± 0.1456*	*5.44*	*77.4*	*0.022*
Log(E2)	0.0128 ± 0.0536	0.05	10.1	0.820	0.0096 ± 0.0255	0.14	77.4	0.707
Log(CORT)	−0.0134 ± 0.0319	0.18	9.7	0.683	−0.0191 ± 0.0159	1.46	76.3	0.231

Reported values are from hierarchical mixed models with clutch as a random effect. Explanatory variables were fitted as both the mean value for a clutch and as centered values (actual value − clutch mean), rendering estimates for between and within clutches, respectively

*ddf* denominator *df*

^a^All numerator *df* = 1

^b^ *P* values are type III values for individual tests, all of which were greater than 0.05

^c^ *P* values are type III values for individual tests. FDR was controlled in a family consisting of the tests of within-clutch relationships for each steroid (in this table and the interaction between last egg and incubation delay). All significant individual tests also had *FDR ≤ 0.05* (indicated in italics)

Within clutches, DHT was negatively correlated with the mean temperature on the day(s) preceding laying of each egg and CORT with the temperature on the day preceding laying of each egg (Table 5, Fig. 2b, d). The other three hormones were not significantly associated with temperature within clutches (Table 6).

**Table 6:** Concentration (pg/mg yolk) of DHT in the yolks of great tit eggs in relation to incubation delay and its interaction with the last egg

	Estimate ± SE	*F* (d*df* ^a^)	*P* ^b^
Intercept	13.654		
Last^b^ egg	−0.226 ± 0.908	0.06 (78.2)	0.804
Incubation delay	0.497 ± 0.499	0.99 (9.1)	0.345
Last^b^ egg × incubation delay	*2.186 ± 0.732*	*8.92* (*78.4*)	*0.004*
(Incubation delay)^2^	−0.127 ± 0.085	2.24 (9.3)	0.168
Last^b^ egg × (incubation delay)^2^	*−0.255 ± 0.124*	*4.24* (*78.4*)	*0.043*

Reported values are from hierarchical mixed models with clutch as a random effect. All listed terms were included in the final model. *P* values (type III) for individual tests of last egg × incubation delay with clutch as a random effect and last egg, incubation delay and last egg × incubation delay as fixed effects were as follows: Log(A4) = 0.161, Log(T) = 0.520, DHT = 0.005, Log(E2) = 0.534, and Log(CORT) = 0.082. FDR was <0.05 for the *P* value for DHT when controlled in a family consisting of the tests of within-clutch relationships for DHT (within-clutch relationships in Table 5 and the interaction between the last egg and incubation delay). Italicized values indicate *P* (type III) < 0.05

*ddf* denominator *df*

^a^All numerator *df* = 1

^b^ *P* values are type III

^c^Last has the value 1 for eggs that are last laid in a clutch and 0 for other eggs

### Yolk hormones in relation to the onset of incubation

Hormones associated with the termination of ovulation and the onset of incubation in the mother’s blood circulation may be able to diffuse or be transported from the follicle or blood circulation into the yolk. Because incubation rarely begins before the completion of laying, the concentration of such hormones may differ in the last egg relative to the rest of the clutch. Hormones associated with the onset of incubation may additionally depend on the interval between clutch completion and the onset of incubation (*incubation delay*; negative values of this variable indicate that incubation began before clutch completion), because hormones in the last egg would reflect earlier *sampling* relative to the onset of incubation. We tested this by additionally fitting terms for the *last egg* and the interaction of last egg with incubation delay into the models describing variation with laying sequence. The only hormone for which this interaction term was significant was DHT (Table 6). Relative (to the rest of the clutch) DHT in last eggs had a quadratic relationship with incubation delay (Fig. 2c). The significant quadratic interaction term indicates only that this relationship is curvilinear and not necessarily that it is peaked. When the parameter estimates and their standard errors are combined (Pezzullo 2007), the estimated incubation delay at which DHT peaks is 3.518 ± 1.849 (SE) days, giving an upper 95 % confidence limit of 7.141 days. Because this value is above the highest value in the data (7 days), the relationship is not significantly peaked. When the quadratic term and its interaction are excluded from the model, the interaction between the last egg and incubation delay remains positive (last egg × incubation delay: estimate = 0.773 ± 0.266 (SE), *F*_1,79.2_ = 8.45, *P* = 0.005). In other words, DHT in the last egg increases overall with incubation delay.

## Discussion

We found correlations between the concentrations of different hormones both within and between clutches and that all hormones except DHT showed significant variation within the laying sequence: A4 increased significantly across the laying sequence, whereas T and E of last eggs (both higher) and CORT of the first egg (lower) differed from the other eggs of the clutch. We found several correlates of yolk hormone concentrations within and between clutches: DHT in the last egg (in relation to other eggs) showed a positive correlation with incubation delay, and DHT and CORT within clutches were negatively associated with temperature on the day(s) preceding laying. The clutch mean DHT level was higher in eggs of females paired with a young male (although this relationship was no longer significant after controlling FDR). Relationships with ambient temperature and incubation delay have not previously been reported for any yolk hormone in birds.

### Correlations among different hormones

**Fig. 3:**
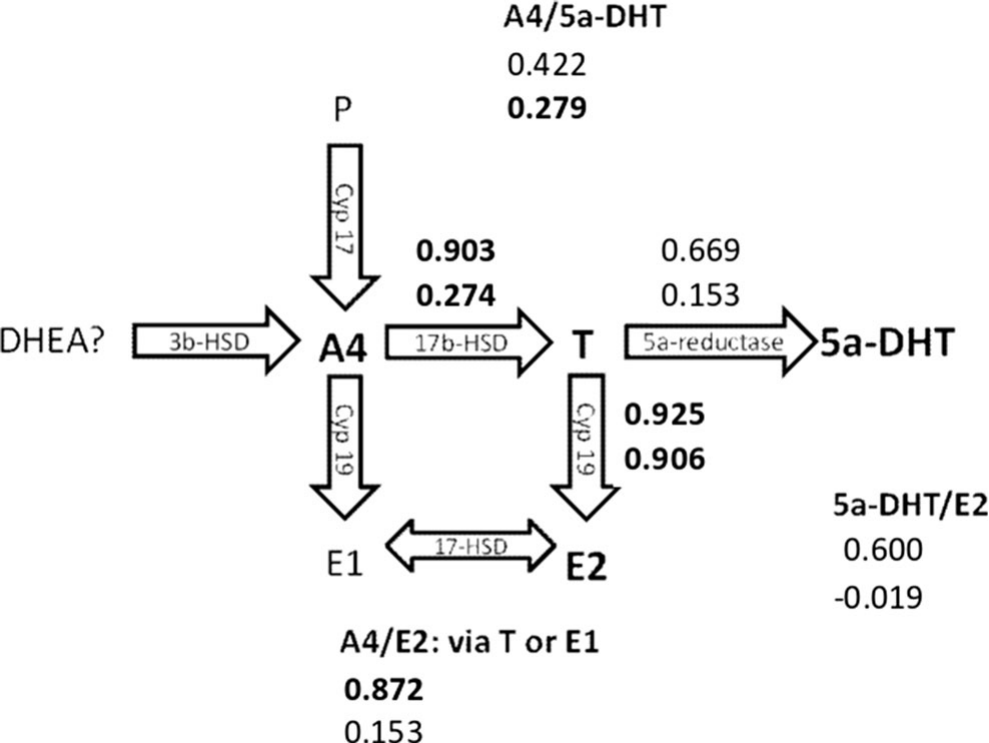
Simplified pathway of follicular steroid synthesis from progesterone (*P*) and dehydroepiandrosterone (*DHEA*) via androstenedione (*A4*) to testosterone (*T*), 5a-dihydrotestosterone (*DHT*), estriol (*E1*), and 17β-estradiol (*E2*). Respective enzymes are indicated in *arrows*: *3b-HSD* 3b-hydroxysteroid dehydrogenase, *17b-HSD* 17b-3b-hydroxysteroid dehydrogenase, *Cyp19* aromatase. Significant correlation coefficients calculated for pairs of steroids at the clutch (*upper value*) and egg (*lower value*) levels are indicated in *bold* (see Table 2)

Among the five steroids that we investigated, T and E2 were strongly correlated both between and within clutches, suggesting that similar physiological mechanisms account for variation in these hormones at both levels. In contrast, the significant differences in the strength of correlations between and within clutches between A4 and T and between A4 and E suggest that different extrinsic or intrinsic factors (including physiological mechanisms) may act between and within clutches. Taken together, we found positive correlations between clutches along the synthetic pathway from A4 to E: A4 and T, A4 and E, and T and E, regulated by 17b-HSD and Cyp19 (aromatase), which catalyze conversion from A4 to T and from T to E, respectively. This suggests that females differ along the pathway of synthesis to E (Fig. 3). A recent study on house sparrows (*Passer domesticus*) found between-female variation in mRNA expression of the Cyp19 enzyme, which could explain the between-female differences in T and E2 (Egbert et al. 2013). The correlation between A4 and T within species is often reported in the literature (but see Schwabl et al. 2007 for contrasting results from intra-specific data), but less is known about correlations with E2. In parallel to our results, the few existing studies on birds and reptiles have also reported positive correlations between E2 and T (Elf et al. 2002; Badyaev et al. 2006; Groothuis et al. 2008; but see Rubolini et al. 2011). In contrast to our results, another study on great tits reports positive correlations between DHT and E2 and also between A4 and CORT (Groothuis et al. 2008).

Our results emphasize that correlations between hormones may differ between within- and among-clutch levels, so data should be analyzed taking into account the grouping of individual eggs into clutches (see also Online Resource 1). Moreover, experimental manipulations of one hormone may disrupt correlations between hormones, with potentially negative effects. Researchers need to be aware of both of these issues.

### Patterns of variation in yolk hormones with the order of laying

Only T (higher than the other eggs) and E2 (higher than the trend through the other eggs) showed significant differences between the last laid egg and other eggs in the clutch. Because any hatching asynchrony tends to be focused on the last egg in great tit clutches, these are the only laying order effects that might plausibly be argued as being adaptively related to hatching asynchrony. The remaining significant laying order effects—a decrease with laying sequence in E2, lower CORT in first eggs, and especially the well-marked increase in A4 across the laying sequence—demand some other explanation. Whether this is facultative adjustment for altering offspring phenotype or a by-product of endocrine processes during egg formation, leading to hormones “leaking” into yolk is not well understood, and we can only speculate about the alternative explanations. For example, plasma E2 levels decrease during laying in other species (Sockman and Schwabl 1999; Williams et al. 2004, 2005), possibly explaining the decreasing yolk E2 pattern in our data. However, it is not clear if the higher (in relation to a linear decrease) E2 levels in the last egg are due to a non-linear trend in the plasma of great tits or to an imperfect correlation between the plasma and yolk E2. In general, evidence from correlations among hormone levels in female circulation and yolk is inconclusive (reviewed in Groothuis and Schwabl 2008). A positive correlation between GnRH-induced rises in plasma T concentrations and yolk T content in some species suggests a potential constraint on the yolk hormone deposition (Jawor et al. 2007; Müller et al. 2011; Peluc et al. 2012). However, although a GnRH challenge causes rapid increases in yolk androgens (A4, T, and DHT) and plasma T, the rises in yolk and plasma T are not necessarily correlated (Egbert et al. 2013).

Previous results from wild great tits from Swiss and Belgian populations show a similarly strong increase in A4 across laying order but also report a linear increase in T (although in the Belgian population, only second and sixth eggs were collected) and decrease in DHT (Tschirren et al. 2004; Heylen et al. 2012). In captive great tits from selection lines, within-clutch patterns of A4 and T were related to personality, and in contrast to our results, no within-clutch patterns in DHT, E2, or CORT were found (Groothuis et al. 2008). We do not know if these differences between populations and studies are related to differences in hatching asynchrony or in environmental conditions such as food supply affecting female physiology or the transfer of hormones into eggs.

### Correlates of between- and within-clutch variation in yolk hormones

In our great tit population, as in most other species, much of the variation in hormone levels occurred between clutches, with repeatabilities similar to previously reported values (Groothuis et al. 2005). We found several significant correlates of between- or within-clutch variation in yolk steroids: first, we found a positive correlation of increased DHT levels in last eggs with incubation delay. There is some evidence for relationships between steroid production and the onset of incubation in American kestrels (Sockman et al. 2001, 2006) and gulls (Müller et al. 2004), possibly mediated via prolactin (Sockman et al. 2006), but clearly this result needs further investigation.

Second, both DHT and CORT were negatively correlated within clutches with temperature preceding laying. The only previous study investigating yolk hormones in relation to ambient temperature found no relationship (Remeš 2011). Additionally, egg mass increased, and composition varied, with ambient temperature in great tits in a Finnish population (Ojanen 1983) but the association between mass and temperature varied across years in the study population (Lessells et al. 2002). The relationships with temperature observed in the current study could simply reflect variation in maternal plasma hormone concentrations, especially for CORT, where low temperatures could be associated with energetic stress caused by lower insect food availability (Wingfield et al. 1997; Jenni-Eiermann et al. 2008). Rapid changes in CORT levels in relation to environmental stressors (e.g., predator encounter, Saino et al. 2005; heat stress, Downing and Bryden 2008; Pitk et al. 2012) have been reported for albumen, but not yolk, as might be expected if CORT was entering the ovulated egg when albumen is deposited in the oviduct. The relationship that we found between CORT in yolk and ambient temperature in the day preceding laying might then be due to passive diffusion from albumen to the yolk.

Third, yolk DHT was higher in the eggs of females paired with a young male (although this difference was not significant after controlling for FDR), a tendency recalling similar findings (for other androgens) in another great tit population (sum of T and A4, Remeš 2011), pied flycatchers (*Ficedula hypoleuca*) (PCA of T and A4, Laaksonen et al. 2011), and collared flycatchers (*Ficedula albicollis*) (T, Michl et al. 2005). One interpretation of relationships between yolk steroids and paternal age is that young fathers are less able or willing to provide parental care (Curio 1983) and that higher yolk DHT levels are a female tactic to elicit more care from these males by enhancing nestling begging (e.g., Müller et al. 2007). This hypothesis has, however, not been generally supported by experimental data or age-specific effects (Tschirren and Richner 2008; Ruuskanen et al. 2009; Barnett et al. 2011; Laaksonen et al. 2011).

Intriguingly, three of the four associations (significant at the level of the individual statistical test, but not, in one case, after controlling for FDR) between steroid levels and the onset of laying or phenotypic or environmental variables were for DHT. The possibility that DHT is particularly responsive to short-term environmental variation is reinforced by the low repeatability and lack of systematic variation through the laying sequence. One could further speculate that in each case, high DHT levels are linked to situations in which the female is more energetically challenged—when she has a longer incubation delay, is laying in low ambient temperatures, or is mated with a young male with a perhaps poorer quality territory. Lower yolk DHT levels (or different within-clutch patterns) have been found in females that were food-supplemented or given a high-quality diet (Verboven et al. 2003; Rutstein et al. 2005; Sandell et al. 2007). However, in another great tit population, total levels of androgens were positively correlated with territory quality and there was no correlation with female condition (Remeš 2011). Unfortunately, we do not have detailed data on female condition and physiology.

In conclusion, we found patterns of yolk hormones both between (male age) and within clutches (temperature preceding laying, interaction between being the last egg and incubation delay), with both of the within-clutch patterns being previously unreported. Whether these are more than statistical artefacts and also found in other contexts (e.g., years, populations and species) requires further study. Intriguingly, most of the patterns were related to DHT rather than A4 and T (which are the most commonly investigated steroids), which suggests that studies concentrating on A4 and T may not reveal the whole story. Our findings of multiple correlates of DHT, the most potent androgen, are complemented by recent experimental studies and comparative analyses that support the idea that DHT has strong effects on offspring development (Schwabl et al. 2007; Hegyi and Schwabl 2010). Thus, the biologically potent steroid DHT clearly deserves more attention.

## Electronic supplementary material

Below is the link to the electronic supplementary material.

ESM 1 (PDF 222 kb)

ESM 2 (XLSX 60 kb)

ESM 3 (PDF 139 kb)
