# Supplementary material for: Yolk steroids in great tit Parus major eggs: variation and covariation between hormones and with environmental and parental factors
Source: Behav Ecol Sociobiol. 2016 Apr 20;70:843–56. doi: 10.1007/s00265-016-2107-1 (PMC4859857; doi:10.1007/s00265-016-2107-1)
Supplement: Supplementary file 1 — (PDF 222 kb) [file 265_2016_2107_MOESM1_ESM.pdf]

Online Resource 1 Pairwise correlations between yolk hormone concentrations

Yolk steroids in great tit *Parus major* eggs: variation and covariation between hormones and with environmental and parental factors
Behavioral Ecology & Sociobiology

C.M.Lessells (1) • S.Ruuskanen (1,2) • H.Schwabl (3)
(1) Department of Animal Ecology, Netherlands Institute of Ecology (NIOO-KNAW), The Netherlands
(2) Department of Biology, University of Turku, Finland
(3) Center for Reproductive Biology, School of Biological Sciences, Washington State University, USA
Corresponding author: *k.lessells@nioo.knaw.nl*

The extrinsic and intrinsic (including physiological mechanisms) factors causing variation in yolk hormones may be shared between different yolk hormones, causing a correlation between concentrations of different hormones across eggs. However, these correlations can be different at the within- and between-clutch level. Such differences are interesting because they imply that different extrinsic and intrinsic factors are at play at those two levels. They also mean that statistical techniques are needed (e.g. mixed models, see Dingemanse and Dochtermann 2013, pp 47-50) that correctly take into account the grouping of eggs from the same clutch, and hence enable the within- and between-clutch correlation to be separately estimated. The absolute minimum sampling requirement for such an analysis is multiple (≥2) eggs from individual clutches.

A visual indication of the between- and within-clutch correlations for a pair of hormones can be obtained by plotting the clutch means, or the individual values centered to the clutch mean, respectively, for the two hormones against each other (see Fig. OR 1.1 for the five steroids investigated in the present study). However, whereas the correlation between the centered values correctly estimates the within-clutch correlation coefficient (see below), **the correlation between the observed clutch means does not correctly estimate the between-clutch correlation coefficient**. This is because the observed clutch means are subject to sampling error and are therefore only estimates of the true (parametric) clutch means. If there is a within-clutch correlation, the sampling errors of the two hormones will be correlated, thus contributing to the correlation between observed clutch means. As a result, the correlation coefficient between clutch means is a weighted average of the between- and within-clutch correlations.

If a single egg has been sampled per clutch, the correlation coefficient between the concentration of hormones x and y,

$$r_{single\ egg\ per\ clutch} = r_{between\text{-}clutch}\sqrt{R_x R_y} + r_{within\text{-}clutch}\sqrt{(1-R_x)(1-R_y)}, \qquad \text{(OR1.1)}$$

where $r_{between\text{-}clutch}$ is the between-clutch correlation coefficient for hormones x and y, $r_{within\text{-}clutch}$ the within clutch correlation coefficient, and R_x and R_y the repeatabilities across eggs within a clutch for the two hormones. $R_{single\ egg\ per\ clutch}$ can also be thought of as the correlation between the clutch means, when the means are based on a sample size of one. Thus the correlation coefficient between clutch means is closer to the between-clutch correlation when the repeatabilities of the two hormones across eggs within clutches is high. The correlation coefficient between the clutch means will also become closer to the between-clutch correlation as the number of eggs sampled per clutch increases. This is because, as the sample size approaches infinity, the observed clutch means approach the true clutch means (i.e. the SEs of the means approach 0). Similarly, when the between- and within- correlation coefficients differ, but a single correlation coefficient is calculated using the values for individual eggs, without recognizing the grouping of eggs within clutches, that estimated correlation coefficient will vary with the number of eggs sampled per clutch.

Table OR1.1 compares the between-clutch correlations (correctly) estimated using mixed models (and reported in Table 2) with the correlations between the clutch means. In each of the 10 comparisons, the correlation between the clutch means is, as expected, intermediate between the between-clutch and within-clutch correlations estimated from mixed models. In general, the discrepancy between the between-clutch correlations and the correlation between the clutch means is not large, but it should be remembered that the discrepancy will increase with decreasing numbers of eggs sampled per clutch (see above). The number of eggs sampled per clutch (between seven and eight on average) in the present study will be (much) larger than the number of eggs sampled in the majority of other studies looking at the correlations between the concentrations of different yolk hormones (if only because of the smaller clutch sizes of most species).

Table OR1.2 compares the within-clutch correlations estimated using mixed models (and reported in Table 2) with the correlations between the centered values (observed value – clutch mean). The correlation coefficient based on centered values does provide an unbiased estimate of the within-clutch correlation coefficient, with the two estimated r values in the present study being within ± 0.002 of each other. The within-clutch correlation coefficient can therefore be obtained from a statistical package calculating the correlation coefficient on the centered values. However, the degrees of freedom assumed by such a package (n – 2, where n is the number of pairs of values) will be incorrect (because it does not account for the degrees of freedom used in centering the values), and hence the *P* value given will be inflated. Instead *P* values can be obtained (from a *P* value calculator etc) using the estimated r value with n_{eggs}-$n_{clutches}$-1 degrees of freedom. This number of degrees of freedom is only strictly correct when the sample size per clutch does not vary. When there is variation in the sample size per clutch the effective degrees of freedom will be further reduced, although, based on a comparison of the *P* values from the mixed model and the correlation coefficient for centered values, the modest variation in eggs sampled per clutch in the present study does not appear to have resulted in appreciably inflated *P* values using the above expression for the degrees of freedom.

In the present study we have estimated the between-clutch correlations between pairs of hormones separately from the within-clutch correlations. In principle, the between-clutch correlation can be further separated into a between-female correlation and within-female-between-clutch correlation. We cannot do this because we have data for only a single clutch per female. Equation OR1.1 can be modified to show that the between-clutch correlation that we have calculated from the mixed models,

$$r_{single\ clutch\ per\ female} = r_{between\text{-}female}\sqrt{R'_x R'_y} + r_{within\text{-}female\text{-}between\text{--}clutch}\sqrt{(1-R'_x)(1-R'_y)}, \qquad \text{(OR1.2)}$$

where $r_{between\text{-}female}$ is the between-female correlation coefficient for hormones *x* and *y*, $r_{within\text{-}female\text{-}between\text{-}clutch}$ the within female between clutch correlation coefficient, and R'_x and R'_y the repeatabilities across clutches within a female for the two hormones.

Readers and authors need to be aware of the level to which a correlation coefficient between hormones refers, and that basing correlation coefficients on means of several individual values rather than single values will alter the value of the correlation coefficient.

References

Dingemanse NJ, Dochtermann NA (2013) Quantifying individual variation in behaviour: mixed-effect modelling approaches. J Anim Ecol 82:39-54. Doi:10.1111/1365-2656.12013

Wikipedia (2014) Student's t test. http://en.wikipedia.org/wiki/Student's_t-test . Accessed 17 July 2014.

Fig. OR1.1 Pairwise correlations between yolk hormone concentrations. Each horizontal pair of plots shows the correlations between one of the 10 pairwise combinations of the five steroid hormones (A4, T DHT, E2 and CORT) analysed. The left-hand column contains the correlations between the clutch mean values for the pair of hormones, and the right hand column the correlations between the centered values (i.e. actual values minus the clutch mean). n = 12 clutches and 93 eggs (except for correlations involving CORT, where n = 12 clutches and 91 eggs). Correlation coefficients corresponding to the points shown in these plots, and between-clutch and within-clutch correlation coefficients derived from mixed models on the same dataset, are given in Tables OR1.1 and OR1.2.

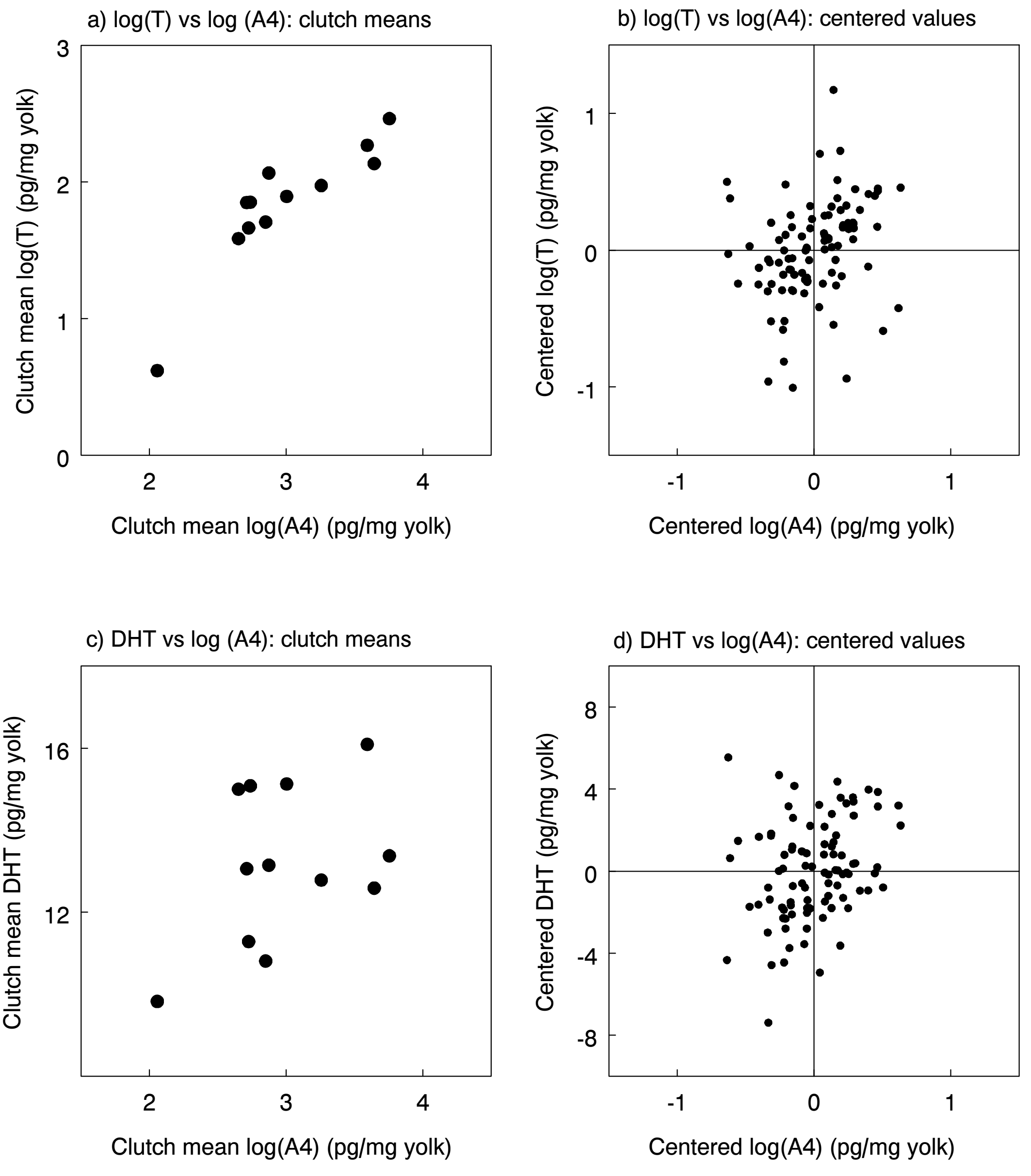

/continued…

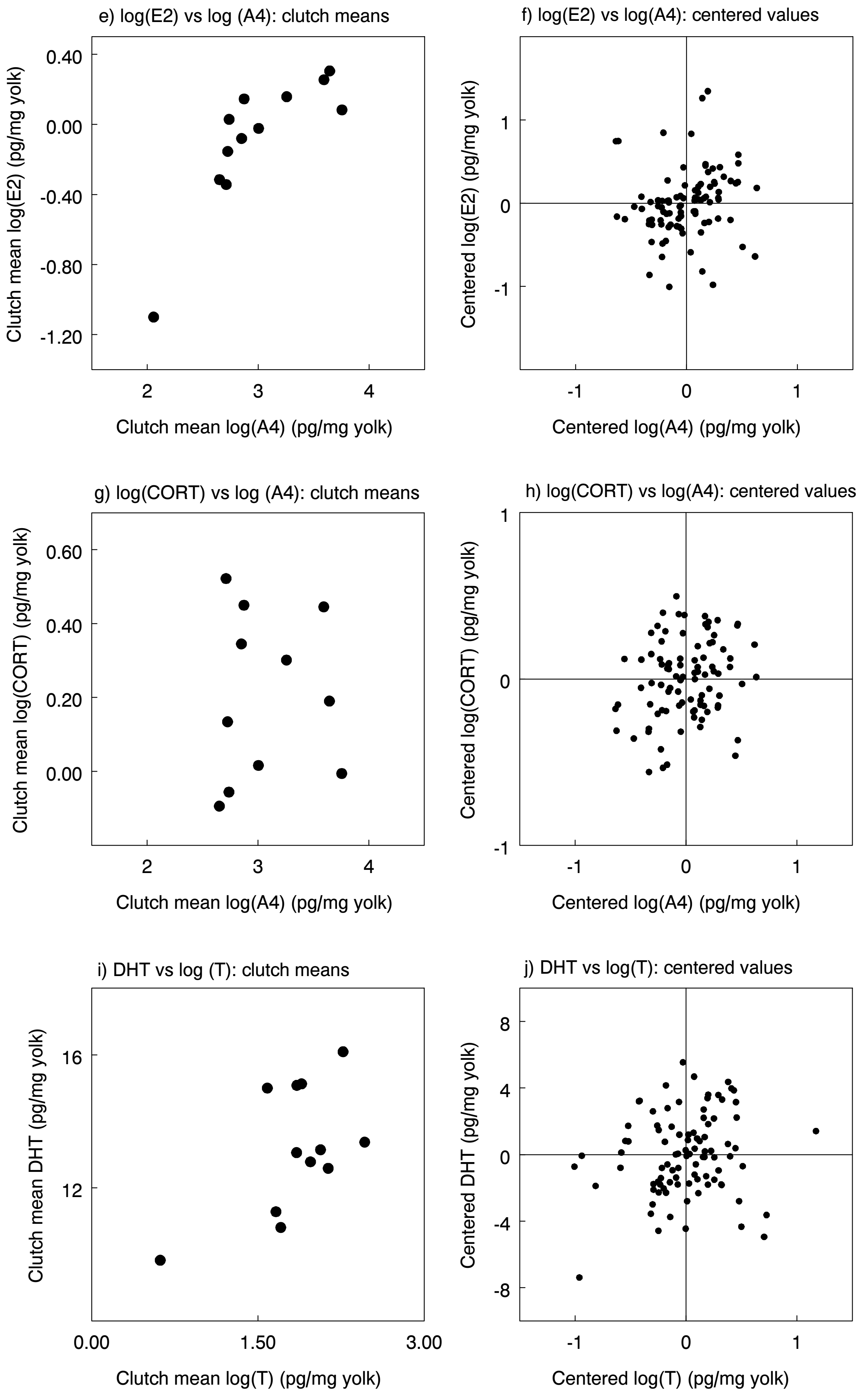
e) log(E2) vs log (A4): clutch means
Clutch mean log(E2) (pg/mg yolk)
0.40
0.00
-0.40
-0.80
-1.20
2
3
4
Clutch mean log(A4) (pg/mg yolk)
f) log(E2) vs log(A4): centered values
Centered log(E2) (pg/mg yolk)
1
0
-1
-1
0
1
Centered log(A4) (pg/mg yolk)
g) log(CORT) vs log (A4): clutch means
Clutch mean log(CORT) (pg/mg yolk)
0.60
0.40
0.20
0.00
2
3
4
Clutch mean log(A4) (pg/mg yolk)
h) log(CORT) vs log(A4): centered values
Centered log(CORT) (pg/mg yolk)
1
0
-1
-1
0
1
Centered log(A4) (pg/mg yolk)
i) DHT vs log (T): clutch means
Clutch mean DHT (pg/mg yolk)
16
12
0.00
1.50
3.00
Clutch mean log(T) (pg/mg yolk)
j) DHT vs log(T): centered values
Centered DHT (pg/mg yolk)
8
4
0
-4
-8
-1
0
1
Centered log(T) (pg/mg yolk)

/continued...

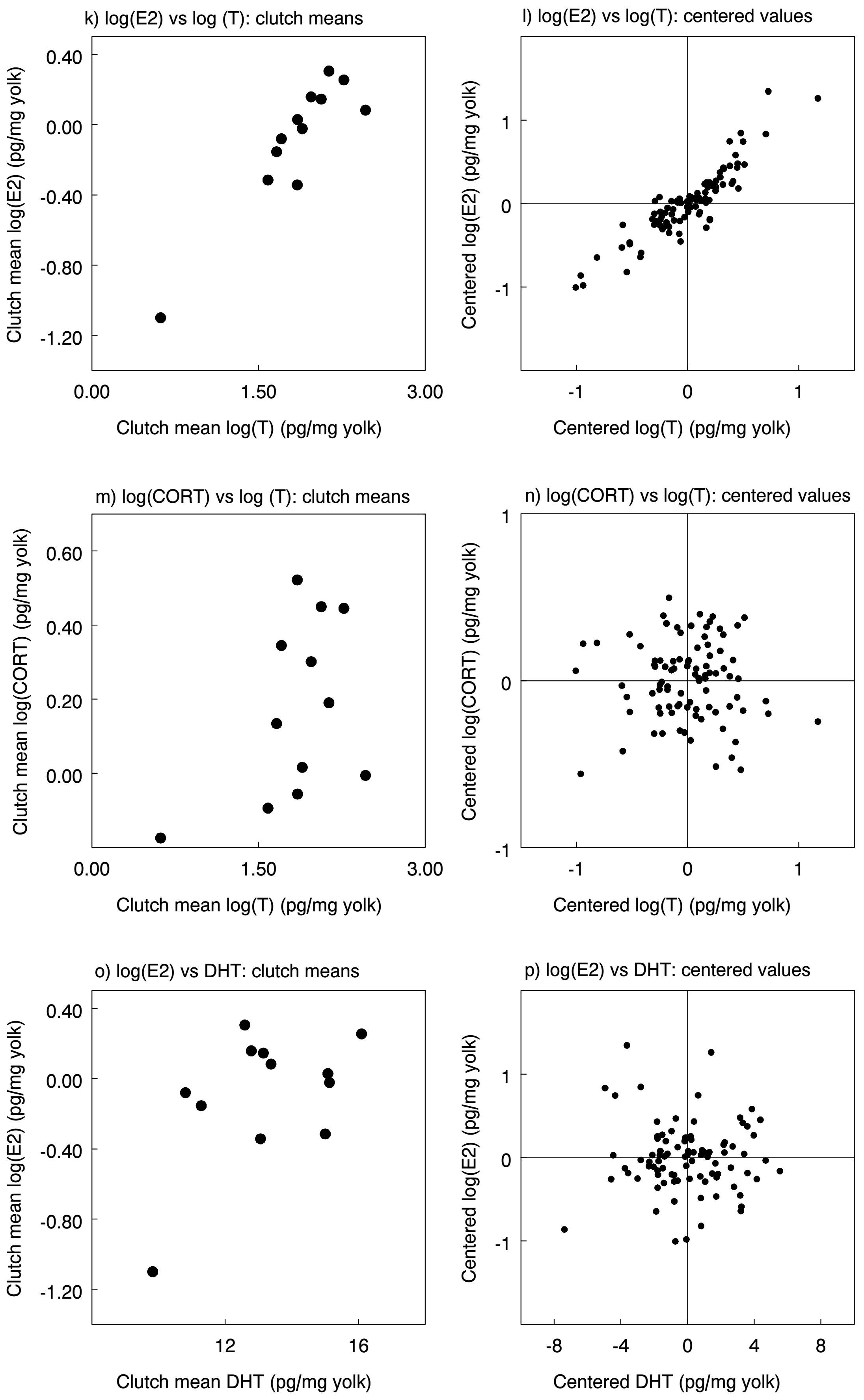
k) log(E2) vs log (T): clutch means
Clutch mean log(E2) (pg/mg yolk)
0.40
0.00
-0.40
-0.80
-1.20
0.00
1.50
3.00
Clutch mean log(T) (pg/mg yolk)
l) log(E2) vs log(T): centered values
Centered log(E2) (pg/mg yolk)
1
0
-1
Centered log(T) (pg/mg yolk)
m) log(CORT) vs log (T): clutch means
Clutch mean log(CORT) (pg/mg yolk)
0.60
0.40
0.20
0.00
Clutch mean log(T) (pg/mg yolk)
n) log(CORT) vs log(T): centered values
Centered log(CORT) (pg/mg yolk)
Centered log(T) (pg/mg yolk)
o) log(E2) vs DHT: clutch means
Clutch mean log(E2) (pg/mg yolk)
12
16
Clutch mean DHT (pg/mg yolk)
p) log(E2) vs DHT: centered values
Centered log(E2) (pg/mg yolk)
-8
-4
0
4
8
Centered DHT (pg/mg yolk)

/continued…

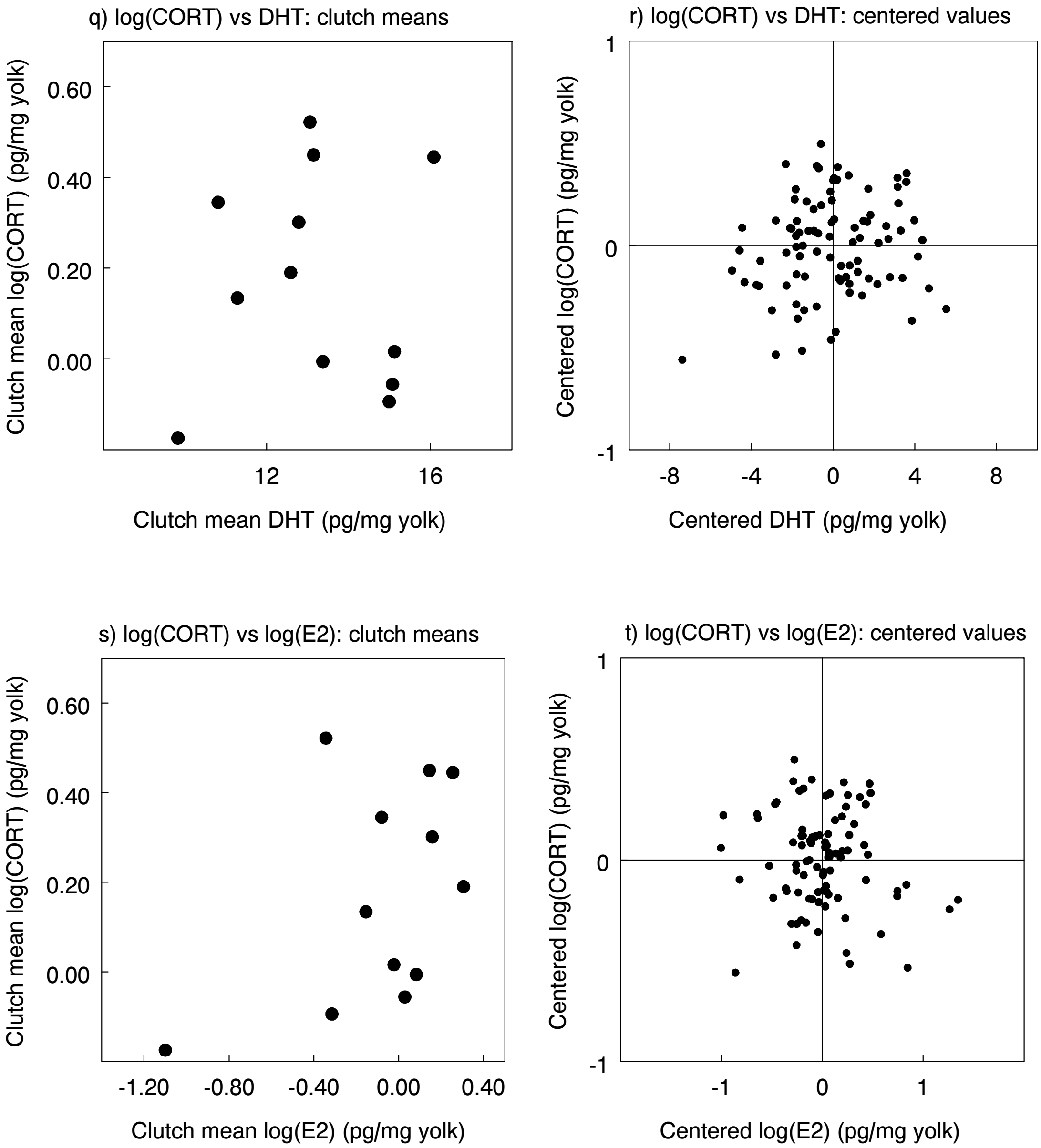
q) log(CORT) vs DHT: clutch means
Clutch mean log(CORT) (pg/mg yolk)
0.60
0.40
0.20
0.00
12
16
Clutch mean DHT (pg/mg yolk)
r) log(CORT) vs DHT: centered values
Centered log(CORT) (pg/mg yolk)
1
0
-1
-8
-4
0
4
8
Centered DHT (pg/mg yolk)
s) log(CORT) vs log(E2): clutch means
Clutch mean log(CORT) (pg/mg yolk)
0.60
0.40
0.20
0.00
-1.20
-0.80
-0.40
0.00
0.40
Clutch mean log(E2) (pg/mg yolk)
t) log(CORT) vs log(E2): centered values
Centered log(CORT) (pg/mg yolk)
1
0
-1
-1
0
1
Centered log(E2) (pg/mg yolk)

Table OR1.1 Between-clutch correlations between A4, T, DHT, E2 and CORT in the yolk of great tit eggs.

Between clutch r([a]*P*)
(upper values are from a mixed model[b], lower values are the Pearson correlation coefficient between clutch means[c])
(n=12 clutches and 93 eggs, except for correlations involving CORT, where n = 12 clutches and 91eggs)

	A4	T	DHT	E2
T	**0.903(<0.001)**			
	**0.889(<0.001)**			
DHT	0.422(0.273)	0.669(0.065)		
	0.418(0.176)	0.572(0.052)		
E2	-([d])	0.925(0.025)[ns]	0.600(0.061)	
	**0.833(<0.001)**	**0.917(<0.001)**	0.522(0.082)	
CORT	0.315(0.061)	**0.502(0.009)**	0.025(0.752)	0.531(0.043)[ns]
	0.310(0.327)	0.463(0.129)	0.050(0.877)	0.460(0.132)

[a] *P* values are from individual statistical tests. FDR was controlled in separate families consisting of the ten *P* values for the correlations from mixed models, and the ten *P* values for the correlations between clutch means. [ns] Indicates FDR > 0.05.

[b] see Methods. *P* values are from likelihood ratio tests based on χ^2 with 1 *df*.

[c] NB: The Pearson correlation coefficients between the clutch means are **not** unbiased estimates of the between clutch correlation.

[d] r and *P* value for the likelihood ratio test are missing because estimation stopped after too many likelihood estimations.

Table OR1.2 Within-clutch correlations between A4, T, DHT, E2 and CORT in the yolk of great tit eggs.

Within clutch r([a]*P*)
(upper values are from a mixed model[b], lower values are the Pearson correlation coefficient based on centered values[c])
(n=12 clutches and 93 eggs, except for correlations involving CORT, where n = 12 clutches and 91eggs)

	A4	T	DHT	E2
T	**0.274(0.011)**			
	**0.273(0.013)**			
DHT	**0.279(0.010)**	0.153(0.168)		
	**0.279(0.011)**	0.153(0.170)		
E2	0.153(0.168)	**0.906(<0.001)**	-0.019(1.000)	
	0.155(0.164)	**0.906(<0.001)**	-0.021(0.851)	
CORT	0.210(0.058)	-0.043(0.655)	0.179(0.107)	-0.118(0.294)
	0.210(0.061)	-0.043(0.705)	0.177(0.116)	-0.119(0.293)

[a] *P* values are from individual statistical tests. FDR was controlled in separate families consisting of the ten *P* values for correlations from mixed models, and the ten *P* values for correlations between centred values. All individual *P* values < 0.05 remained significant after controlling FDR.

[b] see Methods. *P* values are from likelihood ratio tests based on χ^2 with 1 *df*.

[c] *P* values are derived from the r value based on centered hormone concentrations with n_{eggs}-$n_{clutches}$-1 *df*.

Table OR1.3 T tests of the difference between the pairwise correlations between clutch means and between centered values of A4, T, DHT, E2 and CORT in the yolk of great tit eggs.

t (*df*) *P*[a]

	A4	T	DHT	E2
T	**3.415(23.19)0.002**			
DHT	0.453(12.96)0.658	1.486(13.90)0.161		
E2	**3.276(19.18)0.004**	0.082(12.98)0.936	1.860(13.67)0.086	
CORT	0.312(12.86)0.760	1.674(13.48)0.118	-0.379(12.62)0.711	1.914(13.42)0.078

[a] Statistical values are for the difference between the correlation coefficient between the clutch means and the correlation coefficient between the centered values of pairs of hormones using a t test for unequal variances (Welch's t test) with the degrees of freedom estimated using the Welch-Satterthwaite method (Wikipedia 2014). P values are given for the reported *df* rounded down to the next integer. The purpose of this test was to investigate whether there was a difference between the between- and within-clutch correlations between pairs of hormones. The use of the correlation between clutch means as a measure of the beween-clutch correlation is conservative in this context as it is a weighted average of the between- and within- clutch correlations. *P* values are from individual statistical tests. FDR was controlled for the family of ten *P* values. Both individual *P* values < 0.05 remained significant after controlling FDR.
