# Supplementary material for: Yolk steroids in great tit Parus major eggs: variation and covariation between hormones and with environmental and parental factors
Source: Behav Ecol Sociobiol. 2016 Apr 20;70:843–56. doi: 10.1007/s00265-016-2107-1 (PMC4859857; doi:10.1007/s00265-016-2107-1)
Supplement: Supplementary file 3 — (PDF 139 kb) [file 265_2016_2107_MOESM3_ESM.pdf]

Online Resource 3 Concentrations of yolk steroids in relation to the mass of the egg and egg components

Yolk steroids in great tit *Parus major* eggs: variation and covariation between hormones and with environmental and parental factors
Behavioral Ecology & Sociobiology

C.M.Lessells (1) • S.Ruuskanen (1,2) • H.Schwabl (3)
(1) Department of Animal Ecology, Netherlands Institute of Ecology (NIOO-KNAW), The Netherlands
(2) Department of Biology, University of Turku, Finland
(3) Center for Reproductive Biology, School of Biological Sciences, Washington State University, USA
Corresponding author: *k.lessells@nioo.knaw.nl*

Table OR3.1 Concentrations (pg/mg yolk) of A4, T, DHT, E2 and CORT in the yolks of great tit eggs in relation to the mass of the egg and egg components

	Between clutches				Within clutches			
	b ± SE	*F*	[a]*ddf*	*P*	*b* ± SE	*F*	[a]*ddf*	*P*[b]
Explanatory variable: egg mass (g)								
Log(A4)	0.533 ± 1.140	0.22	10.1	0.650	0.442 ± 0.623	0.50	79.1	0.480
Log(T)	0.718 ± 1.063	0.46	10.1	0.515	-1.088 ± 0.786	1.92	79.1	0.170
DHT	5.577 ± 4.204	1.76	10.3	0.213	4.363 ± 5.202	0.70	79.1	0.404
Log(E2)	0.329 ± 0.877	0.14	10.3	0.715	-1.420 ± 0.886	2.57	79.2	0.404
Log(CORT)	-0.718 ± 0.530	1.83	9.8	0.206	0.465 ± 0.522	0.79	76.8	0.377
Explanatory variable: wet yolk mass (g)								
Log(A4)	8.661 ± 7.501	1.33	10.1	0.275	0.042 ± 1.770	0.00	80.1	0.981
Log(T)	9.510 ± 6.825	1.94	10.1	0.194	-3.919 ± 2.291	2.93	80.1	0.091
DHT	17.44 ± 30.37	0.33	9.9	0.579	-6.73 ± 15.56	0.19	80.0	0.667
Log(E2)	5.502 ± 5.919	0.86	10.2	0.374	-2.651 ± 2.582	1.05	80.3	0.308
Log(CORT)	2.862 ± 3.685	0.60	9.7	0.456	0.988 ± 1.506	0.43	77.8	0.514
Explanatory variable: wet albumen mass (g)								
Log(A4)	0.421 ± 1.276	0.11	10.1	0.748	0.844 ± 0.775	1.19	79.0	0.280
Log(T)	0.598 ± 1.195	0.25	10.1	0.628	-0.905 ± 0.988	0.84	79.1	0.362
DHT	6.029 ± 4.702	1.64	10.3	0.228	7.792 ± 6.471	1.45	79.1	0.232
Log(E2)	0.240 ± 0.980	0.06	10.3	0.811	-1.610 ± 1.110	2.10	79.2	0.151
Log(CORT)	-0.887 ± 0.577	2.37	9.8	0.156	0.515 ± 0.656	0.62	76.8	0.435
Explanatory variable: dry shell mass (g)								
Log(A4)	1.793 ± 17.482	0.01	10.3	0.922	-0.816 ± 10.433	0.01	74.1	0.938
Log(T)	7.302 ± 16.915	0.19	10.5	0.675	9.288 ± 12.388	0.48	74.1	0.520
DHT	82.58 ± 66.90	1.52	11.2	0.242	-53.99 ± 85.15	0.40	74.3	0.528
Log(E2)	3.994 ± 13.879	0.08	11.0	0.779	-0.995 ± 15.197	0.00	74.3	0.948
Log(CORT)	-14.362 ± 8.157	3.10	10.0	0.109	20.343 ± 8.523	5.70	71.6	0.020[ns]

[a]*ddf* = denominator *df*; all numerator *df* = 1

Reported values are from hierarchical mixed models with clutch as a random effect. Explanatory variables were fitted as both the mean value for a clutch and as centered values (actual value – clutch mean), rendering estimates for between and within clutches, respectively.

[b] *P* values are from individual statistical tests. FDR was controlled for the family of twenty within-clutch *P* values. [ns] Indicates FDR > 0.05 for *P* values ≤ 0.05
